# Breath-Actuated Nebulizers for Asthma and Chronic Obstructive Pulmonary Disease Exacerbation: A Monte Carlo Simulation Demonstrating National Cost Savings and Length of Stay Reduction

**DOI:** 10.1016/j.acepjo.2025.100112

**Published:** 2025-04-04

**Authors:** Andrew D. Luo, DaMarcus E. Baymon, Gregory A. Peters, Joshua M. Kosowsky, Lauren M. Nentwich, Joshua J. Baugh, Christopher W. Baugh

**Affiliations:** 1 Brigham and Women’s Hospital, Department of Emergency Medicine, Harvard Medical School, Boston, Massachusetts, USA; 2 Massachusetts General Hospital, Department of Emergency Medicine, Harvard Medical School, Boston, Massachusetts, USA

**Keywords:** asthma exacerbation, COPD exacerbation, nebulizer, breath-actuated, cost savings, Monte Carlo simulation, length of stay reduction

## Abstract

**Objectives:**

Breath-actuated nebulizers (BANs) deliver medication only during inspiration, and prior studies have demonstrated their increased efficacy for asthma and chronic obstructive pulmonary disease (COPD) exacerbations compared to continuous nebulizers. However, the widespread adoption of BAN has been limited by its higher per-unit cost. Our primary objective was to estimate the annual national net cost and emergency department (ED) bed-hour savings of switching to BAN for pediatric and adult patients presenting to the ED with asthma or COPD exacerbation.

**Methods:**

We estimated the prevalence of ED visits for asthma and COPD requiring nebulizer treatment using publicly available datasets. We created a Monte Carlo model and ran 1000 trials to determine the marginal cost of a BAN-first approach. We modeled the cost savings from decreased ED bed hours and averted inpatient admissions among eligible patients nationally and by common annual ED visit volumes.

**Results:**

Adoption of a BAN-first strategy nationally is estimated to incur an additional $6,059,000 (±$1,024,000) in supply costs, with total savings of $744,610,000 (±$141,922,000) and a reduction of 178,000 (±77,000) ED bed hours. An ED with 30,000 annual visits would save $206,000 (±$38,000) annually with a supply cost of $1400 (±$260). For 60,000 visits, savings would be $551,000 (±$99,600) with a supply cost of $3700 (±$680). At 130,000 visits, savings would reach $896,000 (±$168,000) with a supply cost of $5900 (±$1100).

**Conclusion:**

BAN may yield significant cost savings driven primarily by a decreased likelihood of admission for COPD exacerbation. Further research is needed to validate clinical efficacy and address barriers to adoption.

**The Bottom Line**

Breath-actuated nebulizers (BANs) are more effective than continuous nebulizers for asthma and chronic obstructive pulmonary disease exacerbations but have higher costs. This study estimated the national financial impact and emergency department (ED) bed-hour savings of switching to BAN. Using public data and a Monte Carlo model, we estimated that the national adoption of BAN would add $6 million in supply costs but potentially save $744 million from prevented hospitalizations and reduce ED bed hours by 178,000 annually. Savings vary by ED size, with significant potential reductions in admissions for chronic obstructive pulmonary disease exacerbations. Further research is needed to confirm clinical efficacy and overcome barriers to adoption.

## 1 Introduction

### 1.1 Background

Respiratory conditions represent a significant proportion of cases seen in the emergency department (ED), accounting for nearly 12 million visits in the United States annually.^1^ For conditions such as asthma and chronic obstructive pulmonary disease (COPD), nebulized medications are a core element of the treatment bundle for patients likely to need an extended ED evaluation or hospitalization. Most nebulizers used in the ED generate aerosol continuously, expelling droplets into the environment during each exhalation and between each breath. The design of these continuous nebulizers (CNs) may result in suboptimal drug delivery into the lungs, wasted medication into the surrounding atmosphere, and possible staff exposure to patient medications and respiratory pathogens.^2^^,^^3^ These challenges are particularly exacerbated in an era of ED overcrowding, with patients routinely receiving care in hallways and communal spaces.

### 1.2 Importance

Breath-actuated nebulizers (BANs) offer an alternative to traditional CNs by releasing aerosol only during the inhalation phase. This mechanism is designed to improve the dose of inhaled medication and lessen environmental contamination.^4^ For asthma and COPD specifically, prior studies have demonstrated that BANs offer several advantages in clinical outcomes, such as a decreased time to symptom resolution, a decrease in ED length of stay (LOS), and a reduction in the overall rate of hospitalizations after ED presentation.^5^, ^6^, ^7^ These effects may have a greater impact in a pediatric asthma population, with a reduction in LOS by nearly an hour due to improved symptom resolution.^8^ Moreover, because aerosol release is limited to inspiration, BAN use may have ancillary benefits, such as reduced disease transmission to hospital staff.^9^ However, to date, the adoption of BANs has been limited, with 1 potential reason being the higher per-unit cost of the BAN apparatus compared to traditional continuous-flow nebulizers.^10^

### 1.3 Goals of This Investigation

In this study, we assess the total cost and potential annual net savings achievable via widespread BAN adoption. We also estimate the associated reduction in ED LOS and avoidable hospital admissions that may be achieved from widespread BAN adoption in the ED. Lastly, we evaluate potential cost savings and throughput impacts for EDs of various patient volumes to characterize these effects at the department level.

## 2 Methods

### 2.1 Selection of Participants

**Figure 1:**
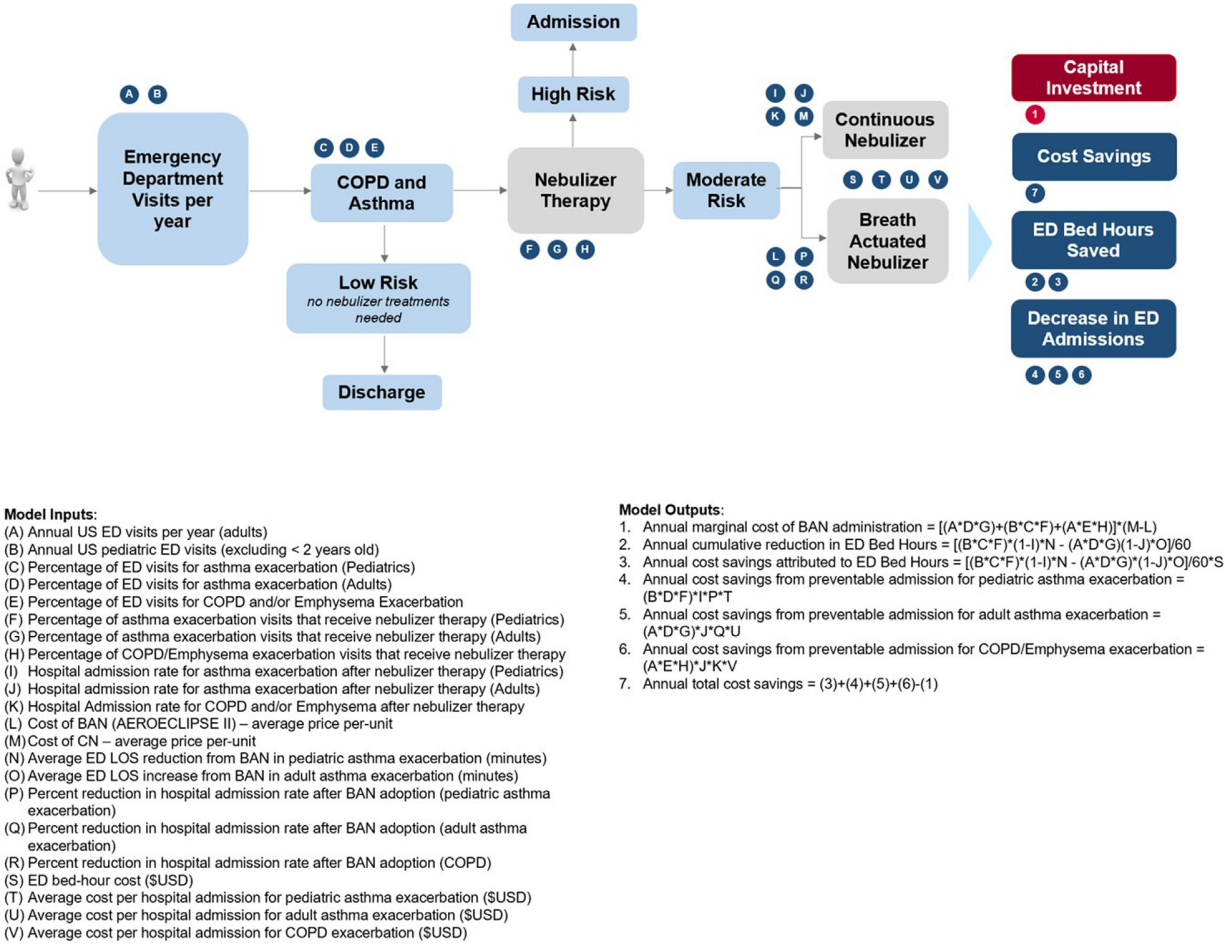
Patient population subdivided into 3 groups, including adult asthma, pediatric asthma (aged 2-18 years), and adult chronic obstructive pulmonary disease (COPD) exacerbations.

Our study population included annual ED visits with (1) a primary diagnosis of asthma or COPD exacerbation and (2) received nebulizer treatment, based on data from the National Hospital Ambulatory Medical Care Survey (NHAMCS). NHAMCS is a national survey conducted by the National Center for Health Statistics, which collects data on ED visits nationally.^1^ We averaged annual ED visits with a primary diagnosis of asthma and COPD exacerbation from 2016 to 2021, to mitigate the risk of variance within any 1 year. The patient population was subdivided into 3 groups: adult asthma, pediatric asthma (aged 2-18 years), and adult COPD exacerbations (Fig 1).

### 2.2 Model

**Figure 2:**
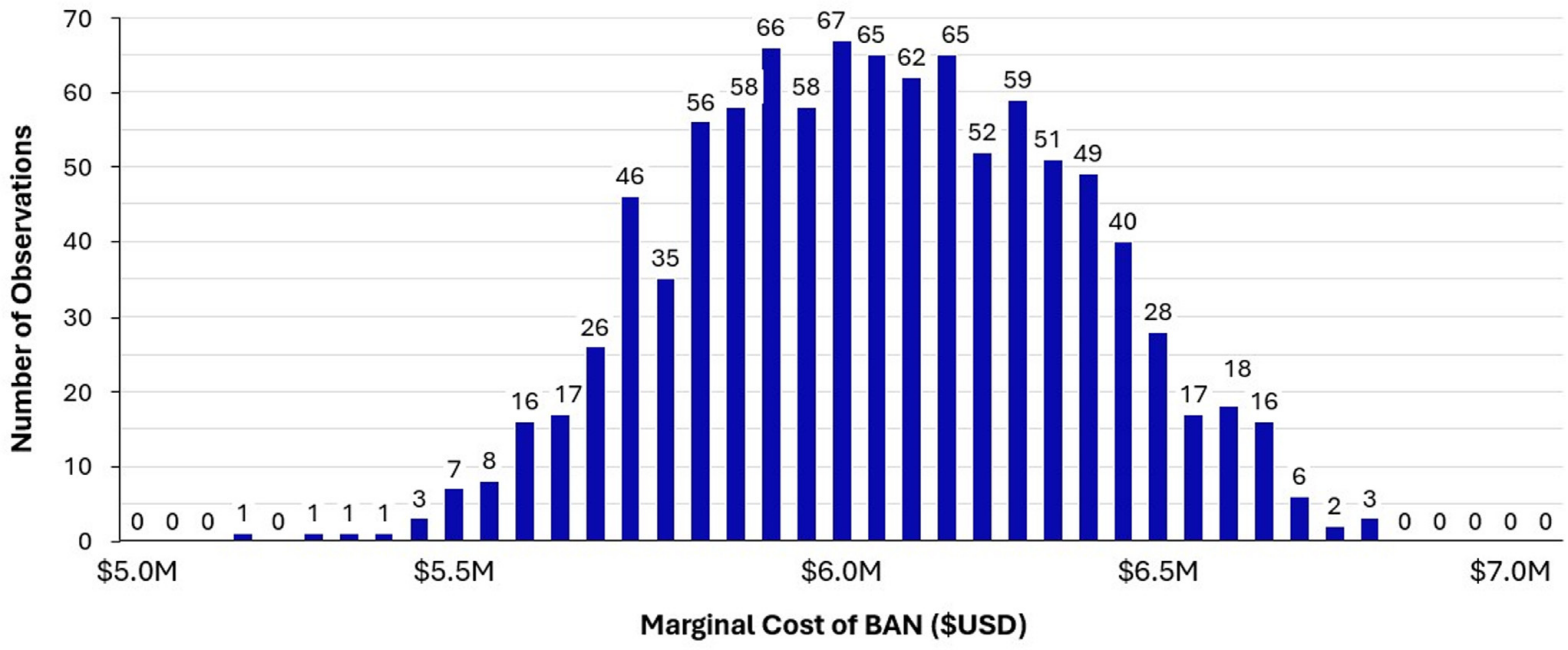
Monte Carlo simulation demonstrating marginal cost of breath-actuated nebulizer (BAN) ($USD).

**Figure 3:**
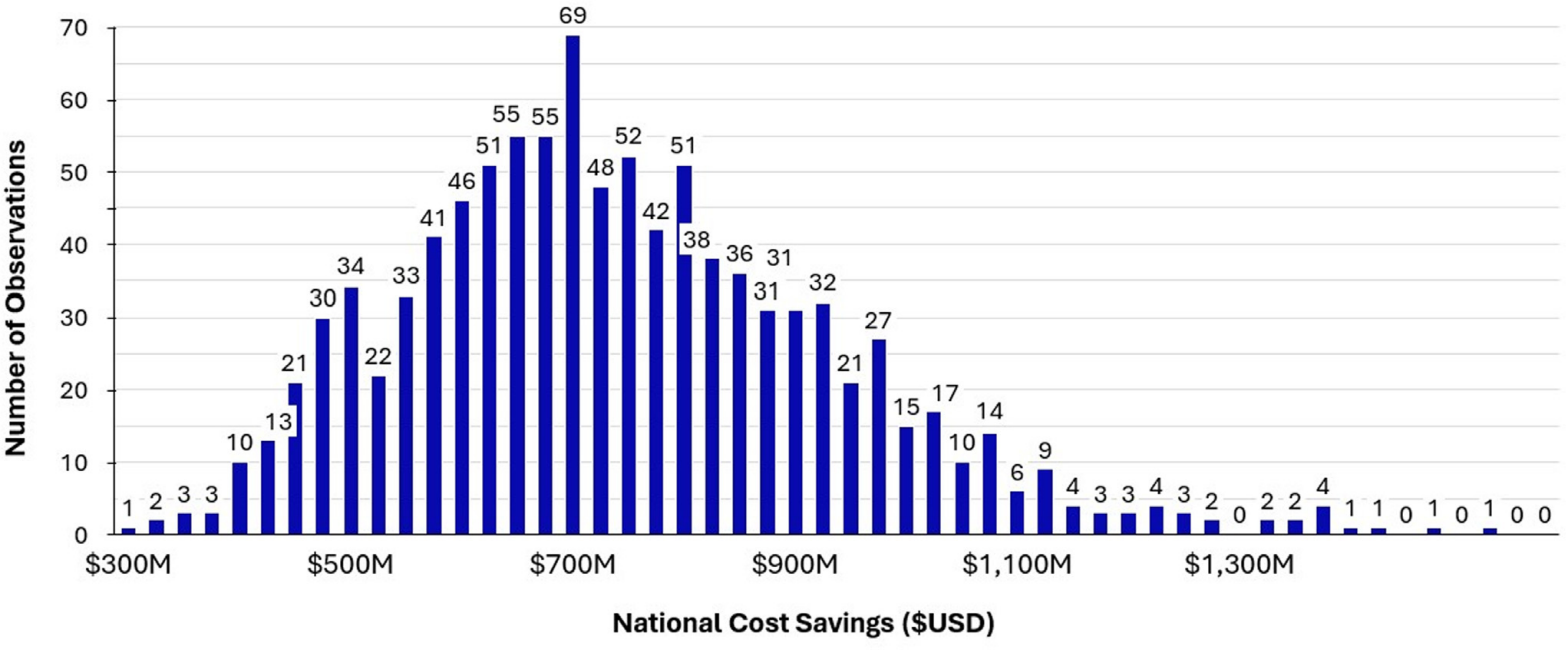
Monte Carlo simulation demonstrating national cost savings from breath-actuated nebulizer (BAN) utilization ($USD).

**Figure 4:**
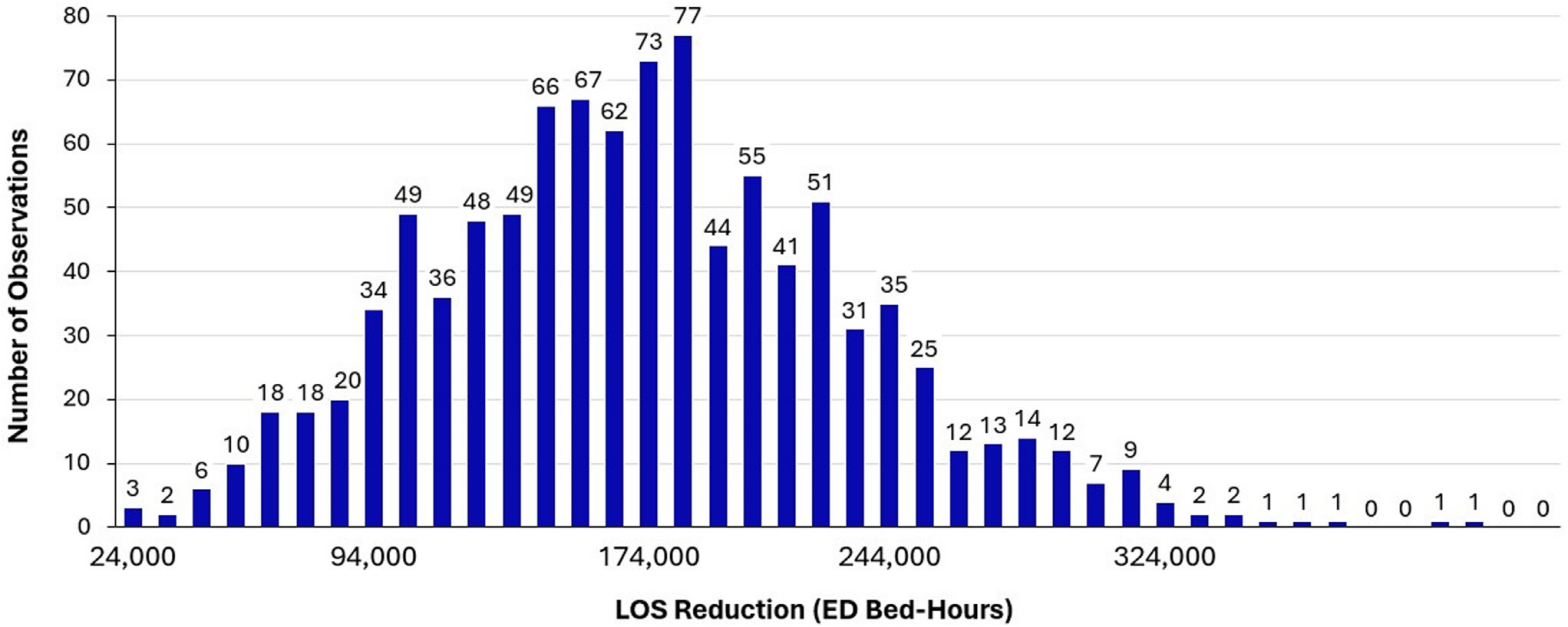
Monte Carlo simulation demonstrating national length of stay (LOS) reduction from breath-actuated nebulizer (BAN) utilization (bed-hours).

We developed a Monte Carlo simulation model to estimate the annual marginal cost of BAN implementation, ED LOS reduction, national health care cost savings, and preventable admissions from BAN usage from the ED. A Monte Carlo simulation is a computational algorithm that uses repeated random sampling for every parameter derived from data distributions. The results of these iterations are then averaged, providing representations of each outcome variable with a distribution, mean, and standard deviation of the final estimate, thereby addressing the uncertainty of the parameters. We conducted 1000 iterations of our model, using input parameters derived from the most recent data distributions available in the literature (Figure 2, Figure 3, Figure 4).

We used Crystal Ball (Release 11.1.2.4, Oracle Corporation) for the Monte Carlo analysis. This study is an analysis of publicly available data and was exempt from institutional board review at our institution.

### 2.3 Model Parameters

The costs associated with BAN adoption included the direct cost of BANs compared to CN, hospitalization costs for asthma and COPD exacerbations, and average ED LOS bed-hour cost. We estimate BAN per-unit cost from industry assessments of nebulizer pricing by IQVIA’s Medical Device and Purchasing Data, which provides information on pricing ranges and volume of nebulizer units sold across different product types.^10^

We assumed that a single nebulizer was used per-ED patient visit and that no additional direct nursing cost would be associated with delivering BAN versus CN. Unless otherwise stated, we assumed a normal distribution for all inputs with reported SD with a 10% CI. For parameters where a range was provided, we assumed a Beta Program and Evaluation Review Technique (BetaPERT) distribution for inputs associated with an interquartile range or 95% CI. BetaPERT distributions are smooth distributions characterized by minimum, most likely, and maximum values. Accordingly, they are most appropriate for describing variables with a reported range of values, such as reported per-unit prices of nebulizers. The marginal cost was estimated by modeling the difference between per-unit cost of BAN and CN using the BetaPERT distribution. Lastly, for LOS estimates, we used a lognormal distribution to capture the skewness and variability commonly seen in conditions such as COPD and asthma exacerbations, in which most patients tend to have similar LOS before being admitted or discharged, while a subset of patients might require longer care, resulting in a right-skewed distribution^11^ (Table 1).^1^^,^^7^^,^^8^^,^^10^^,^^12^, ^13^, ^14^, ^15^, ^16^, ^17^

**Table 1:** Model inputs.

Variable	Description	Value (Mean)	SD	Distribution type	Source	References
A	Annual US ED visit volume (adults)	111,000,000	11,000,000	Normal	NHAMCS 2016-2021	CDC^1^
B	Annual US ED visits (pediatrics excluding <2 years old)	22,000,000	2,200,000	Normal	NHAMCS 2016-2021	CDC^1^
C	Percentage of ED visits for asthma exacerbation (pediatrics)	2.20%	0.22%	Normal	NHAMCS 2016-2021	Primary analysis
D	Percentage of ED visits for asthma exacerbation (adults)	0.86%	0.10%	Normal	NHAMCS 2016-2021	Primary analysis
E	Percentage of ED visits for COPD and/or emphysema exacerbation	0.94%	0.10%	Normal	NHAMCS 2016-2021	Primary analysis
F	Percentage of asthma exacerbation visits that receive nebulizer therapy (pediatrics)	66%	6.60%	Normal	NHAMCS 2016-2021	Primary analysis
G	Percentage of asthma exacerbation visits that receive nebulizer therapy (adults)	57%	5.00%	Normal	NHAMCS 2016-2021	Primary analysis
H	Percentage of COPD/emphysema exacerbation visits that receive nebulizer therapy	41%	4.10%	Normal	NHAMCS 2016-2021	Primary analysis
J	Hospital admission rate for asthma exacerbation after nebulizer therapy (pediatrics)	7.70%	0.70%	Normal	NHAMCS 2016-2021	Primary analysis
K	Hospital admission rate for asthma exacerbation after nebulizer therapy (adults)	5.76%	0.58%	Normal	NHAMCS 2016-2021	Primary analysis
L	Hospital admission rate for COPD and/or emphysema after nebulizer therapy	41.2%	4.12%	Normal	NHAMCS 2016-2021	Primary analysis
M	Cost of BAN: AEROECLIPSE II (average per-unit price)	$5.45	$5.43-$9.07	BetaPERT	IQVIA medical device and supply purchase data	IQVIA Database Q1 2014^10^
N	Cost of CN (average per-unit price)	$0.75	$0.69-$1.83	BetaPERT	IQVIA medical device and supply purchase data	IQVIA Database Q1 2024^10^
O	Average ED LOS reduction from BAN in pediatric asthma exacerbation (minutes)	59	29.5	Lognormal	Single-site, prospective, nonrandomized experimental study (159 patients over a 7-mo study period)	Titus et al^8^
P	Average ED LOS increase from BAN in adult asthma exacerbation (minutes)	13.1	1.31	Lognormal	Single-site, prospective, randomized comparative study (54 patients over 20 y old presenting with wheezing and requiring nebulizer therapy)	Parone et al^13^
Q	Percent reduction in hospital admission after converting to BAN (pediatric asthma)	33.3%	3.33%	Normal	Single-site, randomized comparative study (149 patients over a 13-mo study period)	Sabato et al^7^
R	Percent reduction in hospital admission after converting to BAN (adult asthma)	72.0%	7.2%	Normal	Single-site, randomized study (assessment over a 2-y study period, unknown sample size)	Saunders et al^12^
S	Percent reduction in hospital admission after converting to BAN (COPD)	45.6%	4.6%	Normal	Single-site, randomized study (3600 patients over a 2-y period)	Saunders et al^12^
T	Patient bed-hour cost in the ED (average)	$58.20	$5.80	Normal	Cost analysis from a single-site, urban academic center	Schreyer et al^14^
U	Average cost per admission for pediatric asthma exacerbation	$5200	$520.00	Normal	Center for Medicare Medicaid Services	McDermott et al^15^
V	Average cost per admission for adult asthma exacerbation	$6688	$668.80	Normal	HCUP Nationwide Inpatient Dataset	Kaur et al^16^
W	Average cost per admission for COPD/emphysema exacerbation	$6852	$685.20	Normal	Systemic literature review	Rehman et al^17^

### 2.4 Measurement Outcomes

Our outcome measures were the annual marginal cost of BAN adoption and the associated cost impacts with respect to ED LOS reduction and reduced inpatient admissions. We assumed that all patients receiving nebulizer treatment are using CNs at baseline under the current standard of care. Total cost projections of BAN and CN usage, cost of hospital admissions, ED LOS estimates, and subsequent savings were calculated based on data collected from a comprehensive literature review (Table 1, Fig 1).

To extrapolate the relative cost and savings of utilizing BANs, rather than CNs, at a department level, we developed Monte Carlo simulations for small-, medium-, and large-volume EDs at 30,000, 80,000, and 130,000 visits per year, based on national ED volume data.^18^ For each ED size, we estimated the proportion of adults and pediatric patients presenting with COPD and asthma exacerbations to be similar to what is reported within NAHMCS.

### 2.5 Sensitivity Analyses

We performed several sensitivity analyses to account for limitations in our national cost savings model because some of our assumptions may have disproportionally affected our estimate. First, we performed a sensitivity analysis to determine the impact of BAN on the hospital admission rate of COPD exacerbation cases. We did so because our cost savings estimate for COPD admissions was performed at a single institution, which, when applied to our national dataset, had a large impact on cost savings projection.^12^ To mitigate the influence of this variable, we varied the model outcomes by adjusting the admissions rate reduction from a baseline of 45.6% to 22.8% and 13.7%, representing a 50% and 70% change from our original assumption.

Second, we performed a sensitivity analysis to determine the total cost savings assuming variable adoption of BAN. We examined scenarios in which 25%, 50%, and 75% of eligible patients utilized BAN therapy. This approach allowed us to provide a better understanding of the potential range of outcomes and cost implications at different BAN adoption levels.

## 3 Results

Using averages compiled from the 2016 to 2021 NAHMCS database, we estimated the respective annual incidence of US ED visits for pediatric asthma, adult asthma, and adult COPD exacerbations to be 484,000 with 66% of visits involving nebulizer treatments, 955,000 with 57% of visits involving nebulizer treatments, and 1,040,000 with 41% of visits involving nebulizer treatments. Universal BAN adoption across all EDs in the United States, at a marginal cost of $5.17 (±0.54) per unit, resulted in an incremental increase of $6,059,000 (±$1,024,000) in health care spending nationally (Fig 2). The total annual savings from prevented admissions were $744,610,000 (±$141,922,000) across asthma and COPD exacerbations (Fig 3). When categorized by condition, the corresponding cost savings from prevented hospital admissions were $42,630,000 (±$10,870,000), $150,880,000 (±$35,970,000), and $551,100,000 (±$139,780,000) for pediatric asthma, adult asthma, and adult COPD, respectively.

The impact of BAN adoption on ED LOS for asthma exacerbation was a reduction of 178,000 bed hours (±77,000 bed hours), which correlated to an additional savings of $10,360,000 (±$4,670,000) (Fig 4).

On a per-ED basis, adoption of BANs for small-, medium-, and large-volume EDs was associated with marginal costs of $1400 (±260), $3700 (±$680), and $5900 (±$1100), respectively. Total savings from this initial investment were $205,300 (±$37,800), $547,700 (±$100,000), and $896,000 (±$168,000), respectively. Notably, in the base case of the Monte Carlo simulation, the reduction in admissions from COPD exacerbation accounted for approximately 60% of the total cost savings in the model (Table 2).

**Table 2:** Cost savings stratified by typical ED volume.

	Small ED (30,000 visits/y)	Medium ED (80,000 visits/y)	Large ED (130,000 visits/y)
No.of BAN units needed/y	290 (±40)	780 (±100)	1260 (±170)
Marginal cost of BAN ($USD)	$1400 (±$260)	$3700 (±$680)	$5900 (±$1100)
ED LOS reduction (bed-h)	40 (±18)	107 (±43)	174 (±67)
Reduction in hospital admissions for pediatric asthma exacerbation (visits/y)	9 (±2)	25 (±6)	40 (±9)
Reduction in admissions for adult asthma exacerbation (visits/y)	5 (±1)	14 (±3)	22 (±5)
Reduction in admissions for COPD/emphysema exacerbation (visits/y)	18 (±4)	48 (±11)	79 (±18)
Cost savings from avoided admissions for pediatric asthma exacerbation	$48,500 (±$11,700)	$129,400 (±$31,900)	$210,300 (±$53,500)
Cost savings from avoided admissions for adult asthma exacerbation	$34,000 (±$8800)	$90,800 (±22,300)	$147,500 (±$36,400)
Cost savings from avoided admissions for COPD/emphysema exacerbation	$124,200 (±$30,800)	$331,200 (±$81,500)	$538,300 (±$137,000)
Total cost savings	$205,300 (±$37,800)	$547,700 (±$100,000)	$890,200 (±$167,900)

### 3.1 Sensitivity Analyses

Adjusting the reduction in COPD-related hospitalizations from 45.6% to 13.7% decreased the overall national cost savings to $358,962,000 (±$62,970,000), whereas a change to 22.8% decreased savings to $502,675,000 (±$93,850,000) (Table 3).

**Table 3:** Sensitivity analysis based on reduction of admissions for COPD exacerbation.

		Reduction in COPD exacerbation admissions after BAN implementation (%)		
		13.7% (70% adjustment)	22.8% (50% adjustment)	45.6% (base case)
National	Cost savings attributable to a reduction in COPD hospital admission	$165.450,000 (±$40,045,000)	$309,160,000 (±$79,010,000)	$551,142,000 (±$139,780,000)
	Total cost savings	$358,962,000 (±$62,972,000)	502,675,000 (±$93,848,000)	$744,610,000 (±$141,922,000)
Small ED (30,000 visits/y)	Cost savings attributable to a reduction in COPD hospital admission	$37,300 (±$9,300)	$69,700 (±$17,600)	$124,200 (±$31,700)
	Total cost savings	$119,900 (±19,800)	$152,300 (±$26,200)	$206,200 (±$37,800)
Medium ED (80,000 visits/y)	Cost savings attributable to a reduction in COPD hospital admission	$99,500 (±26,700)	$186,000 (±$45,200)	$331,200 (±$81,500)
	Total cost savings	$319,700 (±$64,900)	$406,100 (±$67,200)	$551,400 (±$99,600)
Large ED (130,000 visits/y)	Cost savings attributable to a reduction in COPD hospital admission	$161,700 (±$41,700)	$302,200 (±$76,700)	$538,300 (±$137,000)
	Total cost savings	$519,500 (±$87,400)	$659,900 (±$114,600)	$896,000 (±$167,900)

Similarly, adjusting BAN adoption rates showed different levels of cost and total savings. At a 25% adoption rate, the total number of BAN units needed per year was 310,000 (±51,500) nationally, down from 1,291,400 (±194,700) units for 100% adoption. Total savings decreased to $180,000,000 (±$42,200,000) at a 25% adoption (Table 4).

**Table 4:** Sensitivity analysis based on adoption rate of BAN (%).

		25% Adoption	Adoption rate of BAN		
			50% Adoption	75% Adoption	100% Adoption (base case)
National	No. of BAN units needed/y (SD)	310,000 (±51,500)	640,000 (±96,900)	953,300 (±174,500)	1,291,400 (±194,700)
	Marginal cost of BAN implementation (SD)	$1,635,000 (±$300,000)	$3,260,000 (±$616,000)	$4,918,000 (±$914,000)	$6,100,000 (±$1,000,000)
	Total cost savings (SD)	$180,000,000 (±$42,200,000)	$361,100,000 (±$83,300,000)	$550,962,000 (±$120,660,000)	$744,610,000 (±$141,922,000)
Small ED (30,000 visits/y)	No. of BAN units needed/y (SD)	70 (±10)	150 (±20)	220 (±40)	290 (±50)
	Marginal cost of BAN implementation (SD)	$360 (±$80)	$730 (±$140)	$1100 (±$280)	$1400 (±$260)
	Total cost savings (SD)	$51,300 (±$10,800)	$102,000 (±$22,000)	$153,100 (±$36,400)	$205,300 (±$37,800)
Medium ED (80,000 visits/y)	No. of BAN units needed/y (SD)	190 (±30)	380 (±60)	580 (±110)	780 (±100)
	Marginal cost of BAN implementation (SD)	$1000 (±$200)	$2000 (±$400)	$2900 (±$660)	$3700 (±$680)
	Total cost savings (SD)	$138,000 (±$28,800)	$273,000 (±$57,000)	$405,000 (±$95,900)	$547,700 (±$99,600)
Large ED (130,000 visits/y)	No. of BAN units needed/y (SD)	310 (±50)	630 (±110)	940 (±180)	1,260 (±170)
	Marginal cost of BAN implementation (SD)	$1600 (±$310)	$3200 (±$670)	$4700 (±$1100)	$5900 (±$1100)
	Total cost savings (SD)	$223,200 (±$46,900)	$445,500 (±$94,900)	$658,200 (±$158,300)	$890,200 (±$167,900)

BAN, breath-actuated nebulizer.

## 4 Limitations

There are several important limitations to this study. First, our analysis was a simulation model and thus was limited by model inputs and structure. Although we informed our model variables using the most recent and widely accepted sources, we made assumptions and adjustments regarding model inputs when data were inconsistent or unavailable and used normal distribution when data were not readily available. Eg, in our review of the literature, one study of BAN found minimal effects on hospitalization and ED LOS, whereas others showed a significant decline in LOS and admissions rates for COPD and asthma.^6^^,^^7^^,^^12^^,^^13^ To mitigate some of these data limitations, we created several sensitivity analyses for data that may have disproportionally impacted our results, such as a reduction in the admission rate of COPD exacerbation and our presumed 100% adoption rate of BAN. Although we preferentially selected sources that evaluated a larger population of patients across a longer duration of observation, some of the references used for our analysis were derived from research abstracts, which may be lower quality compared to peer-reviewed papers. These limitations underscore the need for real-world, multicenter studies to confirm the clinical efficacy and LOS impact of BAN.

Another limitation lies in our assumptions surrounding the costs and savings associated with BAN adoption. We considered the increased per-unit price of BAN devices as the primary expense, but other costs, such as inventory, staff training, and administrative expenses, were not accounted for. Cost savings in this study were characterized as avoidable health care expenses (eg, preventable hospital admissions), which are most effectively realized in accountable care organizations or global budget frameworks. In fee-for-service models, hospitals may find it more challenging to capture these savings directly. However, reducing avoidable admissions and saving bed hours is still likely to be associated with cost savings, especially in health systems with limited capacity.

Although this study focused on COPD and asthma as the primary use cases for nebulizer therapy, the application of BAN in other clinical scenarios is an area for further exploration. For example, nebulizers have recently been used in chemical cardioversion of atrial fibrillation.^19^ This treatment option may present significant advantages over traditional methods such as continuous-flow nebulizers, making it a promising avenue for further exploration in patient care. At present, to our knowledge, few studies have investigated other novel conditions for BAN administration, and therefore, we did not feel that there were enough data to model adequately.

This study does not account for alternative treatment modalities to BAN and CN, such as metered-dose inhalers (MDIs) with spacers.^20^^,^^21^ MDIs have been shown to be a cost-effective and efficacious alternative to traditional CN therapy, particularly for pediatric asthma exacerbation. MDIs are most efficacious in preventing ED revisits rather than decreasing admissions from the ED, which is a demonstrated benefit of BAN. To date, no direct comparisons between BAN and MDI have been reported, nor has there been literature surrounding the prevalence of MDI use in the ED setting. Further study is needed to evaluate the direct difference between BAN and MDIs to better approximate cost savings.

Lastly, our projections may not be applicable across all BAN devices. The BAN literature on which we relied exclusively involved the AeroEclipse nebulizer, patented by Monaghan Medical, the sponsor of this study. To date, there are no other BAN options in the market, and it is unclear how the outcomes might differ from other versions of the product.

## 5 Discussion

This study suggests that adopting BANs could enhance ED throughput and reduce overall care costs for asthma and COPD exacerbations. The marginal cost of switching to BANs is under $6000 per ED annually, with a potential return on investment exceeding 100 times. Pediatric ED could benefit from improved throughput due to the impact of LOS in pediatric asthma cases.

As health systems continue to face mounting financial pressures, there may be hesitancy to invest in costlier equipment. Clinicians often lack standardized tools or policies to guide decisions regarding the adoption of new medical technologies. Current guidelines on the use of novel equipment such as BANs tend to emphasize practical considerations related to nursing and respiratory therapy.^22^ Current guidelines on the use of novel equipment such as BANs tend to focus more on the practicalities related to nursing and respiratory therapy. However, with the growing popularity of alternative care pathways, such as hospital-at-home programs and ED observation units for respiratory illnesses, and the development of more targeted interventions for at-risk patients, technologies such as BANs can enhance the standard of care.^23^, ^24^, ^25^, ^26^

Our study underscores the impact of BAN adoption across the entire episode of care and provides a potential foundation for clinicians to advocate for BAN use based on total-cost-of-care savings.^27^ Beyond direct cost savings within an episode of care for asthma or COPD, widespread BAN adoption may also yield societal benefits, particularly in terms of productivity. COPD and asthma affect up to 15% of the adult population, with average hospitalization lengths for these conditions reaching up to 5 days.^28^^,^^29^ In aggregate, the morbidity of asthma and COPD represents significant losses in productive working days for adults and students, which is yet another cost saving that we did not consider in this analysis.^30^ We also did not consider other ancillary benefits associated with BAN use, such as improved patient comfort during treatment, particularly in children, and more rapid symptom resolution.^4^ Future studies should invest these additional advantages of BAN, in conjunction with larger patient trials that confirm the clinical efficacy of BAN.

Overall, this Monte Carlo simulation study suggests that adopting BANs for ED patients with asthma and COPD may yield substantial cost savings from inpatient admission rates. In addition, there may be decreased ED LOS for pediatric asthma exacerbation. Although these findings are promising, further real-world studies are needed to corroborate the simulation results, explore other potential benefits, such as reduced airborne pathogen transmission, and guide the broader implementation of BAN in clinical practice.

## Author Contributions

DEB and CWB conceived the initial project concept. ADL and CWB developed the analytical Monte Carlo model. All authors provided data sources necessary for model inputs and assumptions. ADL and DEB drafted the manuscript, and all authors contributed substantially to its revision, providing important intellectual content.

## Funding and Support

This study was supported by a grant from Monaghan Medical Corporation. Monaghan Medical Corporation had no input into the study design, data analysis, or manuscript review.

## Prior Presentations

American College of Emergency Physicians Conference 2024.

## Conflict of Interest

CWB: paid speaker for Roche Diagnostics, Octapharma, and CE Symmetry, an investigator for Abbott Laboratories, an advisory board participant for Roche Diagnostics, Salix Pharmaceuticals, Pfizer Inc., and AstraZeneca, a consultant for Abbott, Pfizer, Roche, and an advisor to Lucia Health Guidelines.
